# The effect of the arrangements law on patient mix in Israeli private healthcare

**DOI:** 10.3389/fpubh.2025.1674658

**Published:** 2025-11-04

**Authors:** Royi Barnea, Hadar Goldshtein, Adi Niv-Yagoda

**Affiliations:** ^1^Assuta Health Services Research Institute, Tel Aviv, Israel; ^2^School of Health Systems Management at Netanya Academic College, Netanya, Israel; ^3^Department of Economics, Ben-Gurion University of the Negev, Beersheba, Israel; ^4^The Faculty of Medicine & Health Sciences, Tel Aviv University, Tel Aviv, Israel

**Keywords:** health financing, inequality, private hospitals, public funding, socioeconomic status, health policy, Israel

## Abstract

**Introduction:**

Health inequalities remain a persistent challenge, yet little is known about how financing reforms affect access within private hospitals. In 2016 Israel introduced the shorten waiting times reform, designed to expand publicly financed surgeries and reduce reliance on supplementary and private insurance.

**Methods:**

This study examined its impact on both the financing mix and the socioeconomic composition of surgical patients. Administrative data on 1,082,685 procedures performed at Assuta Medical Centers between 2015 and 2019 were analyzed, comparing pre-reform (2015- October 2017) and post-reform (November 2017-2019) periods, with ambulatory procedures serving as a control group.

**Results:**

Publicly financed surgeries increased from 5% to 51% (ATT +51.6pp; 95% CI 43.3-59.9; *p* < 0.001), while supplementary and private financing declined. The share of middle-SES patients rose from 52% to 57% (+7.1pp), high-SES declined from 35% to 29% (−8.0pp), and low-SES increased modestly from 13% to 14% (+0.9pp). In contrast, the control group showed only minimal changes.

**Discussion:**

These findings indicate that the reform was associated with a substantial reallocation of financing and a measurable broadening of SES representation, particularly for middle-income groups, with incremental gains for disadvantaged populations. Overall, the results are consistent with improved equity in access and highlight how regulatory tools can harness private capacity for public benefit within a universal health system.

## Introduction

Inequality in healthcare is a widespread global phenomenon driven by social structures such as income, education, and place of residence (1, 2). These inequalities are reflected in disparities in access to services and health outcomes. For example, in the United States, wealthy individuals live on average 10–15 years longer than poor individuals, largely due to differences in insurance coverage and access to timely, high-quality medical care (3). The World Health Organization has identified equity as a central challenge, calling for reforms that improve the distribution of resources, strengthen service delivery, and ensure access regardless of socioeconomic background (2). The Israeli healthcare system is predominantly public, financed through general taxation and a dedicated health tax, and governed by the National Health Insurance Law of 1994, which established the principles of justice, equality, and mutual aid (4–6). All residents are insured through one of four non-profit health maintenance organizations (HMOs), which are responsible for delivering the services included in the basic health basket. This basket is updated annually and covers hospitalizations, surgeries, medications, and community care, although copayments are required for some services. In addition to the universal coverage, 79% of the population holds supplementary insurance (SHABAN), and a large share also holds private commercial policies (7–10). Israel's combined coverage rate of supplementary and private insurance (~83%) is among the highest in the OECD, compared with an average of about 35% in member states (11). Despite universal coverage, substantial disparities remain across geographic regions and population sectors. Residents of the periphery have fewer hospital beds per capita and live on average 2–4 years less than residents of central areas (12–14). Infant mortality rates are higher in the north and south, while life expectancy and overall health status in the Arab population are lower compared to national averages (15, 16). These disparities reflect both broader socioeconomic conditions and structural gaps in infrastructure, technology, and human resources. In response to these inequities and to growing dependence on private expenditure, the government has implemented several reforms since 2015 aimed at reinforcing the public health system. Three central measures stand out: (i) a reimbursement arrangement that set new rules for the flow of payments between insurers and providers; (ii) the introduction of a cooling-off period restricting physicians from immediately shifting patients between public and private sectors, intended to reduce conflicts of interest; and (iii) the shorten waiting times program, launched in September 2016 (17, 18). The latter was a budgeted initiative designed to expand the supply of elective surgeries by financing them publicly, irrespective of provider ownership, with the dual aims of reducing waiting times and lowering private out-of-pocket spending. Following the implementation of these reforms, early monitoring indicated an increase in the number of publicly funded surgeries performed in private hospitals and a parallel decline in privately financed procedures. However, it remains unclear whether these shifts translated into measurable changes in the socioeconomic composition of patients accessing private hospitals. The present study addresses this gap by examining the impact of the shorten waiting times reform on patients undergoing surgery at Israel's largest private hospital network. Using a quasi-experimental design with ambulatory procedures as an untreated control group, we test two expectations: (1) that the reform shifted the financing of surgeries from supplementary and private sources toward public coverage, and (2) that the socioeconomic composition of surgical patients broadened, with increased representation of middle- and lower-SES groups.

## Methods

The original dataset included over 1.7 million surgical procedures and more than 11 million ambulatory procedures and diagnostic tests (e.g., imaging, endoscopy) performed at Assuta Medical Centers between 2015 and 2022. To isolate the effect of the 2016 shorten waiting times reform, data from 2020 to 2022 were excluded due to the substantial disruptions caused by the COVID-19 pandemic. Including pandemic-era data could have confounded the results and obscured whether observed changes reflected the reform itself or broader system-wide shocks. After excluding those years and applying data cleaning, the final analytic window focused on 2015–2019. The unit of analysis was the surgical episode. Inclusion criteria were all elective surgical procedures recorded in hospital administrative systems during this period. Exclusion criteria included non-surgical encounters, records with missing key fields (date, hospital, procedure type, financing source, or patient locality), and duplicate records (episodes appearing more than once across databases). After cleaning and deduplication, the final surgical sample comprised 1,082,685 procedures: 420,318 performed between January 2015 and October 2017 (pre-reform) and 662,367 performed between November 2017 and December 2019 (post-reform). An additional 7 million ambulatory procedures and diagnostic tests were retained as a control group not targeted by the reform. Financing categories were defined according to the primary payer recorded at the closure of each episode: (i) public funding (Form 17 issued by an HMO), (ii) supplementary insurance (SHABAN), (iii) private commercial insurance, and (iv) out-of-pocket payments. Where multiple payers were recorded, episodes were classified according to the dominant source, with public coverage superseding private contributions in cases of mixed payment. Socioeconomic status (SES) was determined from the Israeli Central Bureau of Statistics index of patients' residential locality, grouped into three categories: low (clusters 1–4), middle (clusters 5–7), and high (clusters 8–10). Comparisons were conducted between pre- and post-reform periods for both surgical and ambulatory control groups. Analyses were carried out in R and Microsoft Excel. We employed a quasi-experimental difference-in-differences (DiD) design with ambulatory procedures as the untreated control domain. Event-study models were estimated to test parallel pre-trends and to capture dynamic effects around the reform's introduction. Statistical significance was defined as *p* < 0.05. For the main DiD estimates, we constructed monthly aggregates by group (treatment, control) and month. The treatment group included surgical procedures, and the control group included ambulatory procedures. The primary outcome was the monthly share of publicly financed surgical episodes, expressed in percentage points, computed as the count of publicly financed episodes divided by the total number of surgical episodes in that month. SES outcomes were defined as monthly shares of patients from low, middle, and high SES localities among surgical episodes. We estimated DiD models on these monthly aggregates using a treatment x post indicator, where post equals 1 from November 2017 onward and 0 otherwise. Aggregate means were weighted by the monthly sample size, and standard errors were clustered at the month level. Event-study specifications were estimated as functions of months relative to the reform month with the pre-reform month −1 as the reference.

## Results

**Figure 1:**
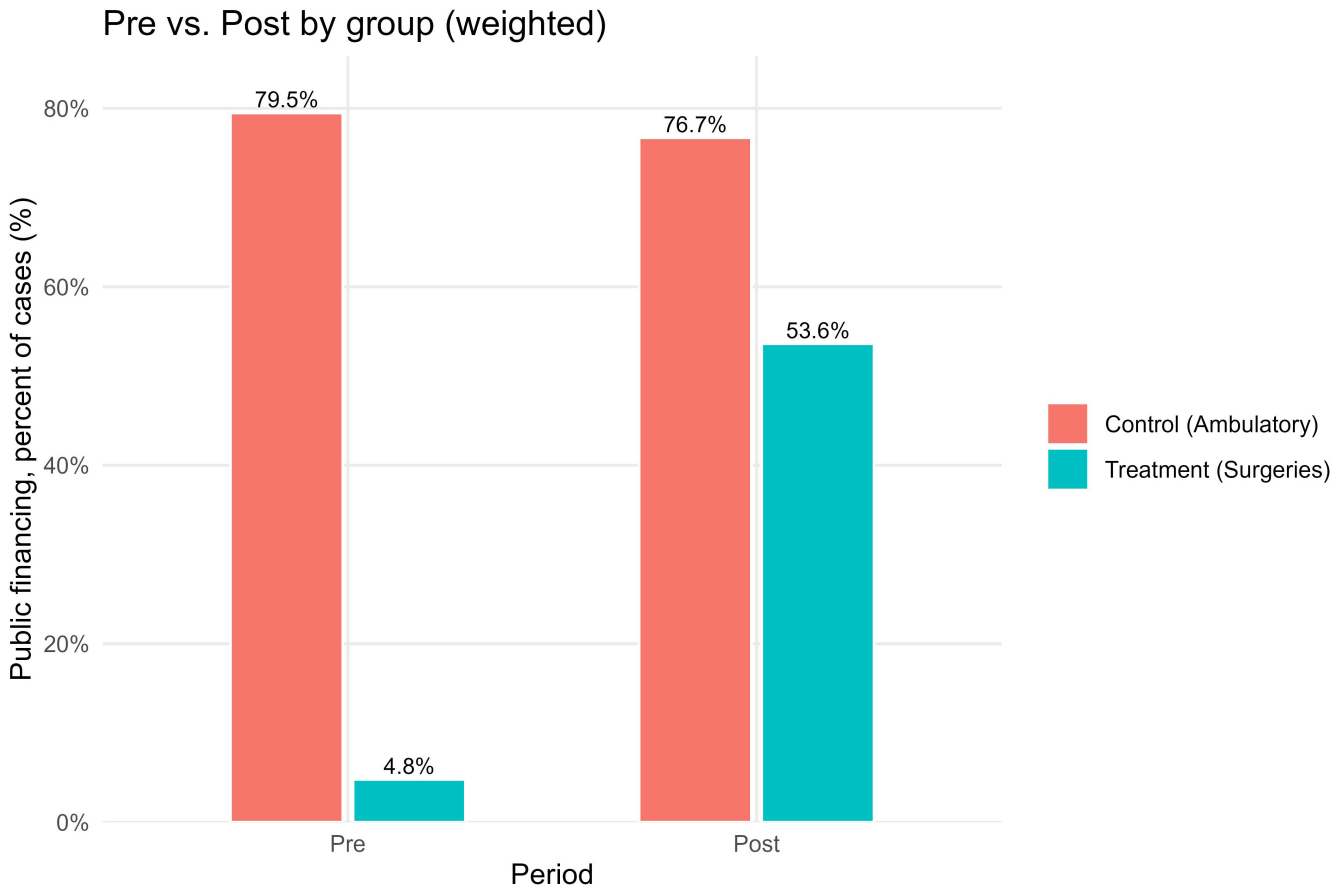
Public financing percent, pre- vs. post-reform, by group (treatment vs. control); weighted means with labels.

**Figure 2:**
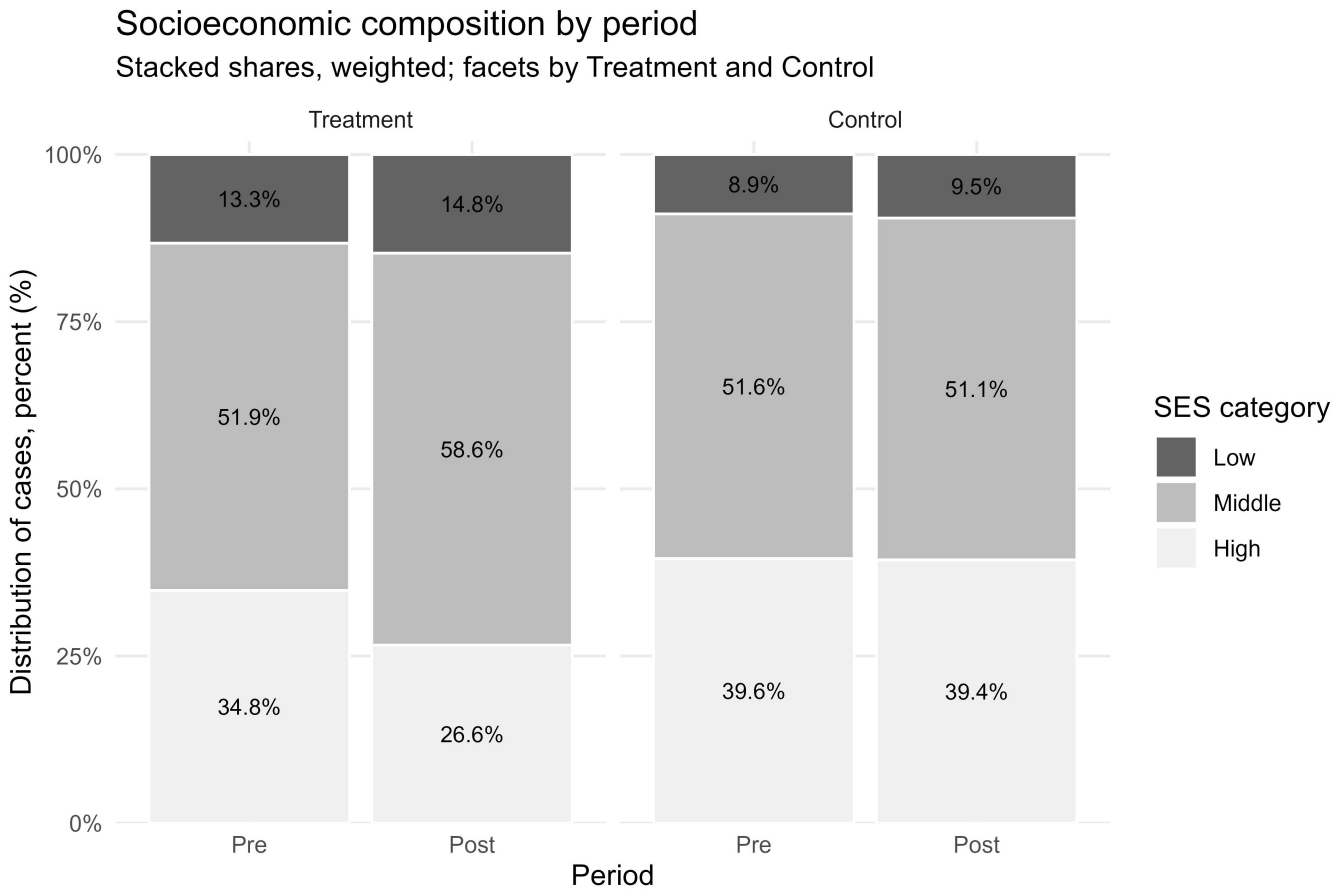
Socioeconomic composition of patients (low/middle/high), pre- vs. post-reform; faceted by treatment and control; stacked percentages with labels.

**Figure 3:**
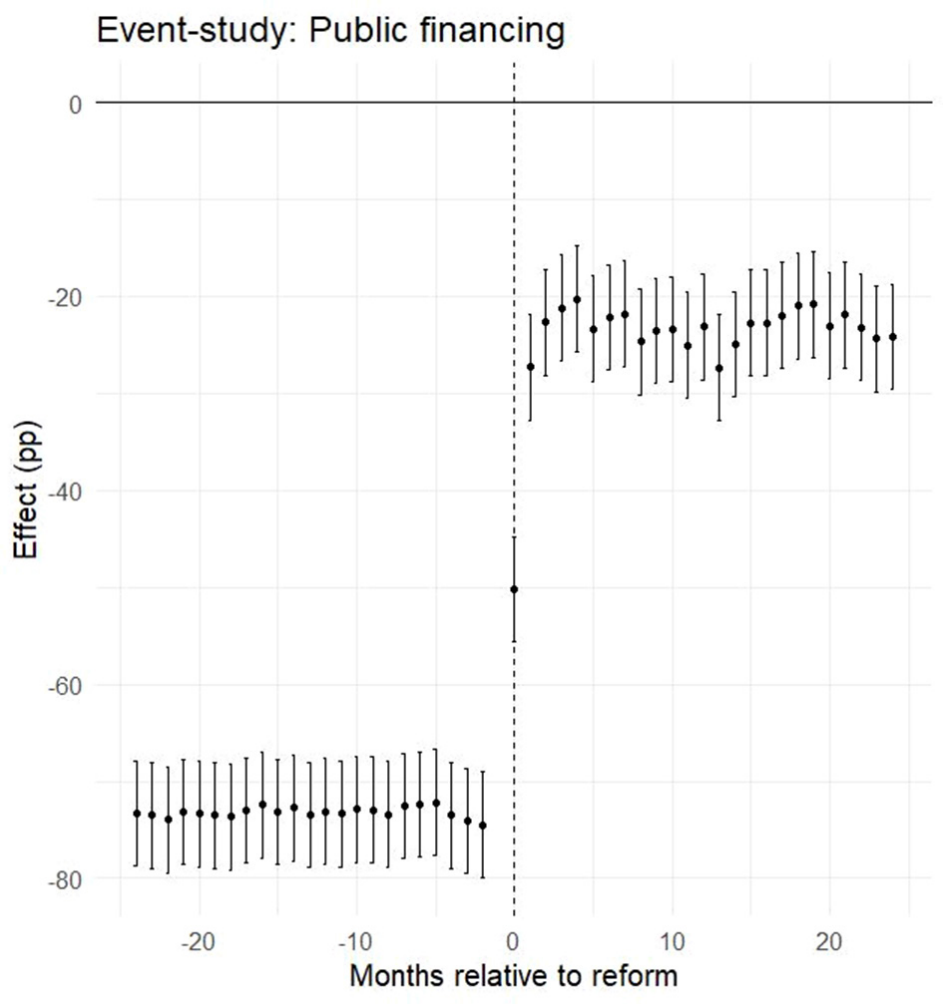
Event-study estimates for public financing (pp): coefficients relative to reform month (*k* = 0) with 95% CIs; parallel pre-trends and discrete post-reform level shift.

**Figure 4:**
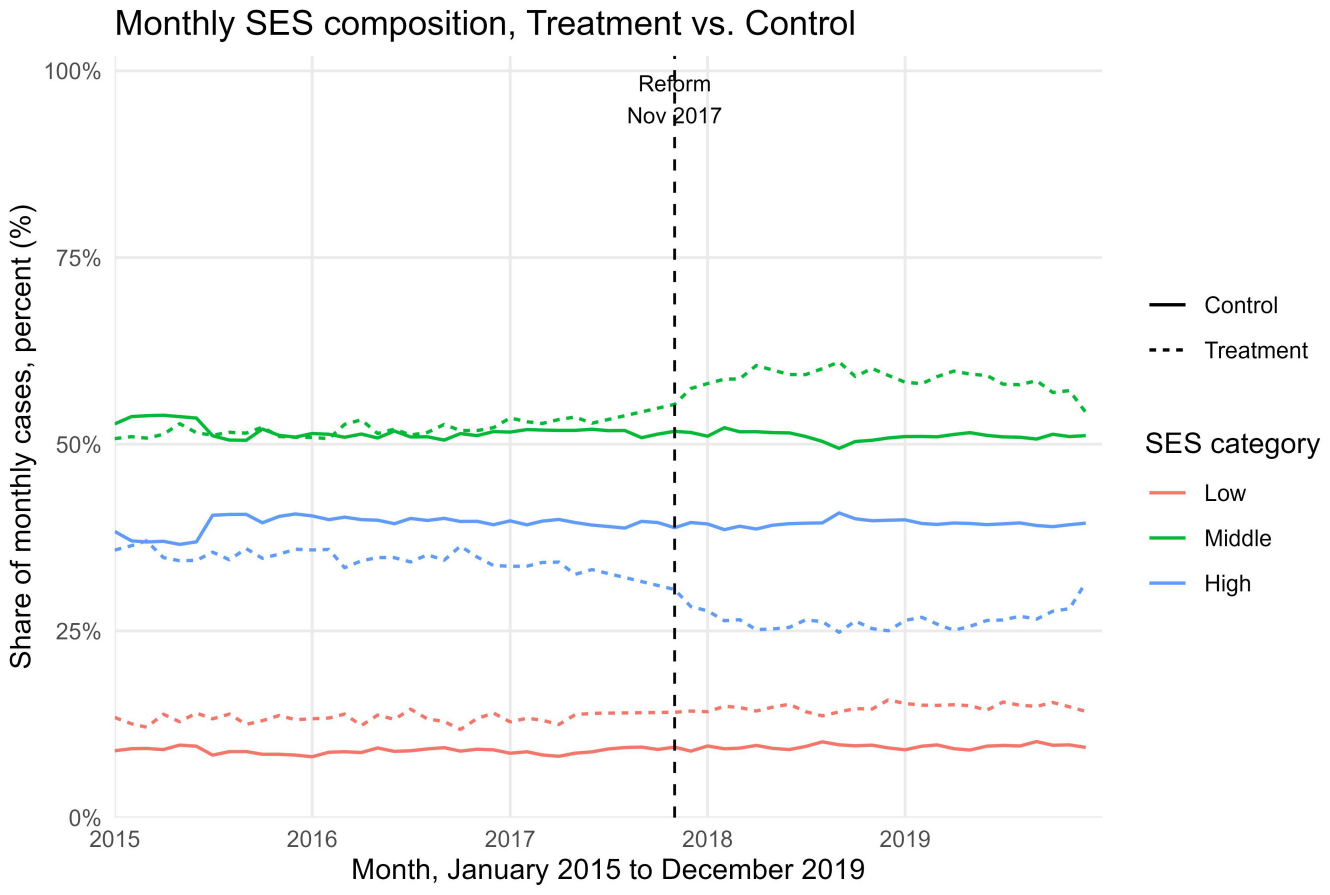
Monthly SES composition (% low/middle/high) over time, treatment (dashed) vs. control (solid); vertical line marks reform month (November 2017).

After excluding pandemic-era years, the final sample included 1,082,685 surgical procedures: 420,318 performed before the reform (January 2015–October 2017) and 662,367 after its implementation (November 2017–December 2019). In addition, ~7 million ambulatory procedures and diagnostic tests were retained as a control group not affected by the reform. The most striking change concerned financing patterns. Prior to the reform, only 5% of surgical procedures were covered by public funds (Form 17), while 95% were financed through supplementary insurance (SHABAN), private insurance, or out-of-pocket payments. After the reform, this distribution reversed: 51% of surgeries were publicly financed, and 49% were privately financed. This represents an average treatment effect of +51.6 percentage points in public financing (95% CI: 43.3–59.9; *p* < 0.001; Table 1). Consistent with these estimates, Figure 1 shows that public financing for surgeries rose from 4.8% in the pre-reform period to 53.6% in the post-reform period, while the ambulatory control remained high and nearly flat (79.5% to 76.7%). Raw group means are provided in Supplementary Table S1. Regarding socioeconomic composition, Table 1 reports a modest increase in the share of low-SES patients (+0.9 pp; 95% CI: 0.5–1.2; *p* < 0.001), a larger increase in middle-SES (+7.1 pp; 95% CI: 6.4–7.9; *p* < 0.001), and a corresponding decrease in high-SES (−8.0 pp; 95% CI: −8.8 to −7.2; *p* < 0.001). Figure 2 shows the stacked shares. In the treatment group, the share of low-SES rose from 13.3% in the pre-reform period to 14.8% post-reform, the share of middle-SES rose from 51.9% to 58.6%, and the share of high-SES fell from 34.8% to 26.6%. In the control group, changes were minimal: low-SES increased slightly from 8.9% to 9.5%, middle-SES changes from 51.6% to 51.1% and high-SES declined from 39.6% to 39.4%. Detailed pre- and post-period distributions are reported in Supplementary Table S2. These results indicate that access to private hospitals has become more socially diverse, primarily due to greater inclusion of middle-income groups, with incremental gains for patients from disadvantaged backgrounds. The ambulatory control group remained virtually unchanged. Event-study estimates confirmed the robustness of these findings. As shown in Figure 3, financing trends between treatment and control groups were parallel in the pre-reform period, supporting the validity of the difference-in-differences design. In the event-study (Figure 3), pre-reform coefficients hover around −70 percentage points with overlapping confidence intervals; at month 0 (November 2017) there is a discrete jump toward roughly −20 to −25 percentage points that persists thereafter, consistent with a sustained level shift. Immediately after the reform, there was a discrete and sustained upward shift in public financing of surgeries that persisted throughout the observation period. Similarly, monthly SES distributions revealed a sustained post-reform re-weighting: a decline in the share of high-SES patients and steady growth in middle-SES representation, while the control group remained stable (Figure 4). In the monthly series (Figure 4), the treatment group's middle-SES share rises from about 52 percent to roughly 58-60 percent, while high-SES falls from about 39 percent to the low-30s; the control series remain relatively flat over the same period. Overall, the results consistently demonstrate that the 2016 shorten waiting times reform substantially changed both the financing structure and the socioeconomic profile of surgical patients in private hospitals. The convergence of large and statistically significant DiD estimates (Table 1), the financing reversal (Figure 1), the redistribution of SES composition (Figure 2), the confirmation of parallel pre-trends (Figure 3), and the time-series SES trends (Figure 4) all point to the same conclusion: the reform expanded the role of public financing and broadened access to private hospital services across a more diverse patient population.

**Table 1:** Difference-in-differences estimates of the reform's effect on surgical patients (treatment group relative to ambulatory controls).

**Outcome**	**ATT (pp)**	**CI 95%**	***p*-value**
Public financing	+51.6	[43.3, 59.9]	< 0.001
Low SES share	+0.9	[0.5, 1.2]	< 0.001
Middle SES share	+7.1	[6.4, 7.9]	< 0.001
High SES share	−8.0	[−8.8, −7.2]	< 0.001

Notes: Average treatment effect on the treated.

Estimates from difference-in-differences models on monthly aggregates with a treatment × post indicator, weighted by monthly sample size; standard errors clustered by month. pp = percentage points. SES categories follow CBS clusters: low = 1-4, middle = 5-7, high = 8-10.

## Discussion and limitations

This study shows that the 2016 shorten-waiting-times reform in Israel was associated with a substantial shift in both the financing and the sociodemographic profile of patients undergoing surgical procedures in a large private hospital chain. Using a difference-in-differences design with ambulatory procedures as a control group, we estimated that the an average treatment effect of +51.6 percentage points in public financing (95% CI: 43.3–59.9; Table 1). Event-study analyses confirmed the absence of differential pre-trends and documented a discrete level shift coinciding with the reform (Figure 3). These findings provide consistent evidence that the reform redirected surgical financing from private and supplementary insurance toward public coverage, in line with its stated objectives. In terms of socioeconomic status, the reform produced a measurable redistribution in the patient mix. The share of patients from low-SES localities increased modestly (+0.9 pp, 95% CI: 0.5–1.2), the representation of middle-SES patients rose more substantially (+7.1 pp, 95% CI: 6.4–7.9), and the proportion of high-SES patients declined accordingly (−8.0 pp, 95% CI: −8.8 to −7.2). (Table 1; Figures 2 and 4; see Supplementary Tables S1-S2 for descriptive pre/post means). These shifts suggest that the reform primarily broadened access for middle-income groups, while also achieving incremental gains in inclusion of patients from disadvantaged backgrounds (Figures 2, 4). The overall pattern reflects a partial rebalancing of access in private hospitals, traditionally dominated by high-income patients, toward a more socially diverse population. The absence of meaningful changes in the control group reinforces the interpretation that the observed effects are attributable to the reform rather than to broader secular trends. These findings should be considered in light of ongoing debates about the role of private hospitals within universal health systems. International evidence shows that publicly financing care in private settings can reduce waiting times and expand capacity, but requires careful regulation to prevent resource diversion and to safeguard equity. Israel's policy innovations, including the introduction of a cooling-off period and restrictions on physician dual practice, reflect such balancing efforts. The results of this study suggest that leveraging private capacity under public financing can enhance access, provided that regulatory safeguards are in place. Long waiting times for medical services in public health systems are a widespread problem around the world (19). In Israel, the National Health Insurance Law requires that medical services be delivered at a reasonable quality and distance from the patient's residence, yet the law does not define what constitutes a “reasonable” waiting time. The absence of this definition, combined with structural shortages in healthcare infrastructure, personnel, and overall public expenditure on health, has exacerbated inequality in access to services (13, 20). These challenges have led many Israelis to purchase private health insurance as a means to avoid long wait times for surgery. However, this trend risks deepening inequality by creating a two-tier system, one for those who can pay and one for those who cannot. Additionally, private insurance may lead to overutilization of services (moral hazard), increase national healthcare spending, and place greater strain on system resources (21). Survey data illustrate these dynamics. A 2021 public opinion survey showed that one-fifth of respondents had foregone medical treatment due to lack of accessibility, and 42% of them subsequently turned to private care. Moreover, 35% had skipped treatment because of long waiting times, with half of them seeking private services instead (16). These findings underscore the growing gap between those who rely on public healthcare and those who can afford private options. Many countries have attempted to reduce health inequalities through structural reforms. For instance, Canada introduced per capita budgeting for provinces in an effort to improve resource allocation, but this approach has raised concerns that it does not adequately reflect variations in population aging, disease burden, and regional disparities (22). In New Zealand, the government reorganized the healthcare system to better serve disadvantaged populations, including the provision of targeted services for addiction, mental health, and disability care, and the creation of dedicated health centers for underserved communities (23). Such examples suggest that reducing inequality requires active efforts to ensure access for populations with low socioeconomic status (24), who are less likely to benefit from private insurance alternatives (25). Another possible solution is to increase the share of publicly financed services at the expense of private expenditure, while promoting technologies and strategies that reduce reliance on human resources and hospitalization infrastructure. This should be complemented by efforts to expand the medical workforce in the short term. Studies have also shown that cooperation between public and private hospitals can be an effective mechanism for reducing waiting lists, with evidence pointing to up to a 60% reduction in waiting times for hospitalization through such partnerships (26). Within Israel's dual system, it is crucial to maintain a balance between public and private sectors. Private providers can help manage waiting times and offer patient choice and quality service, provided that they do not siphon resources away from the public sector. Policymakers must ensure that access to care is not contingent on the ability to pay. Importantly, a distinction should be made between ownership of service provision (public vs. private institutions) and the source of financing (private out-of-pocket vs. public funds). Performing publicly financed surgeries in private hospitals may be a sustainable and efficient strategy to expand access and leverage private capacity for public benefit (17). Reforms in the Israeli healthcare system have increasingly aimed to reinforce the public orientation of services provided within private institutions. This trend reflects a broader policy goal of harnessing private sector capacity to expand access to publicly financed care, while safeguarding equity and avoiding resource misallocation. A key distinction should be made between the ownership of service provision (public vs. private institutions) and the source of financing (public vs. private payment). Publicly financed surgeries performed in private hospitals may improve availability and efficiency, particularly by utilizing existing infrastructure such as operating rooms, and can potentially help reduce waiting times. However, this integration also risks exacerbating socioeconomic disparities if not properly regulated. To address these concerns, recent legislation introduced mechanisms that strengthen the public identity of private providers. For instance, patients are allowed to select their surgeon in a private hospital under public funding, but a “cooling-off” period is required before the same physician can provide the patient with private care. These regulatory tools aim to balance patient autonomy with the system's commitment to equity, ensuring that access to care is not determined by the ability to pay, and that the use of shared resources aligns with public healthcare objectives (27). Our previous research has shown that, following reforms in the Israeli healthcare system, private hospitals have increasingly functioned as providers of publicly financed surgical services (17). Building on this insight, the current study demonstrates a clear relationship between the method of financing and patients' sociodemographic characteristics. In a country marked by significant disparities across sectors and regions, this model, where procedures are supplied privately but funded publicly, may serve as a promising tool for expanding equitable access to care. Limitations must be acknowledged. First, the analysis was restricted to surgical procedures and did not examine long-term outcomes such as health status, waiting times, or patient satisfaction. Second, the data derive from a single private hospital network, albeit the largest in Israel, comprising four hospitals, and may not capture the entire healthcare landscape. Third, although the quasi-experimental DiD and event-study designs strengthen causal interpretation, unobserved system-level changes or shifts in referral patterns may still bias estimates. Finally, while the reform increased representation of middle-income patients, additional policies may be needed to ensure greater inclusion of low-SES populations.

## Conclusion

This study shows that the 2016 waiting time reform in Israel was associated with a major redistribution in both financing sources and the socioeconomic profile of patients undergoing surgery in private hospitals. In Assuta, the largest private network, the share of publicly financed surgeries increased by 51.6 percentage points (ATT; Table 1; Figures 1, 3), while the patient mix shifted toward greater representation of middle-SES groups, alongside a modest but significant increase in low-SES participation and a corresponding decline in high-SES dominance (Figures 2, 4). In contrast, ambulatory procedures not subject to the reform showed no comparable changes, reinforcing the interpretation that the observed effects are policy-driven rather than secular trends. These findings suggest that the reform was consistent with improved equity in access to surgical care, particularly by broadening inclusion of middle-income groups with incremental gains for disadvantaged populations. Despite limitations, including the focus on surgical procedures and reliance on data from a single private hospital network, this study provides large-scale quantitative evidence on the equity effects of structural reforms in Israel's mixed public–private system. For policymakers, the results underscore the value of leveraging private infrastructure under public financing, with appropriate safeguards to prevent resource diversion, as a strategy to expand access, strengthen equity, and enhance the resilience of the national health system.

## Data Availability

The raw data supporting the conclusions of this article will be made available by the authors, without undue reservation.
