# Supplementary material for: The effect of the arrangements law on patient mix in Israeli private healthcare
Source: Front Public Health. 2025 Nov 4;13:1674658. doi: 10.3389/fpubh.2025.1674658 (PMC12623352; doi:10.3389/fpubh.2025.1674658)
Supplement: Supplementary file 1 [file Table_1.docx]

## Supplementary Table S1. Public financing (weighted %)

| **Treatment Pre** | **Treatment Post** | **Δ Treatment** | **Control Pre** | **Control Post** | **Δ Control** | **DiD (ΔT-ΔC)** |
| --- | --- | --- | --- | --- | --- | --- |
| 4.8 | 53.6 | 48.8 | 79.5 | 76.7 | -2.8 | 51.6 |

Notes: Values are weighted percentages. Δ is the within-group change (Post minus Pre). DiD is Δ(Treatment) minus Δ(Control).

## Supplementary Table S2. SES shares (stacked %)

| **SES** | **Treatment Pre** | **Treatment Post** | **Δ Treatment** | **Control Pre** | **Control Post** | **Δ Control** | **DiD (ΔT-ΔC)** |
| --- | --- | --- | --- | --- | --- | --- | --- |
| Low | 13.3 | 14.8 | 1.5 | 8.9 | 9.5 | 0.6 | 0.9 |
| Middle | 51.9 | 58.6 | 6.7 | 51.6 | 51.1 | -0.4 | 7.1 |
| High | 34.8 | 26.6 | -8.2 | 39.6 | 39.4 | -0.2 | -8.0 |

Notes: SES categories are Low (CBS 1–4), Middle (5–7), and High (8–10). Values are stacked shares in percent. Δ is the within-group change. DiD is Δ(Treatment) minus Δ(Control).
